# Investigation of Functional Synergism of CENPF and FOXM1 Identifies POLD1 as Downstream Target in Hepatocellular Carcinoma

**DOI:** 10.3389/fmed.2022.860395

**Published:** 2022-07-05

**Authors:** Daniel Wai-Hung Ho, Wai-Ling Macrina Lam, Lo-Kong Chan, Irene Oi-Lin Ng

**Affiliations:** Department of Pathology and State Key Laboratory of Liver Research, University of Hong Kong, Hong Kong, Hong Kong SAR, China

**Keywords:** CENPF, FOXM1, POLD1, liver cancer, HCC, functional synergism

## Abstract

**Background:**

Lines of evidence implicate CENPF and FOXM1 may have novel co-operative roles in driving hepatocellular carcinoma (HCC).

**Objective:**

We investigated the clinicopathological correlation, functional characterization, molecular mechanism and translational significance of CENPF and FOXM1.

**Methods:**

We carried out integrative studies investigating functional synergism of CENPF and FOXM1 in HCC and its metastasis. Human HCC samples, HCC cell lines and mouse model were used in the studies. Stable knockdown, q-PCR, Western blotting, whole-transcriptomic sequencing (RNA-seq), as well as cell and mouse assays were performed.

**Results:**

Upon clinicopathological correlation, we found that co-overexpression of CENPF and FOXM1 in human HCCs was associated with more aggressive tumor behavior including presence of venous invasion, tumor microsatellite formation, and absence of tumor encapsulation. Moreover, co-silencing FOXM1 and CENPF using shRNA approach in HCC cell lines resulted in significantly reduced cell proliferation. Furthermore, our RNA-seq and differential gene expression analysis delineated that CENPF and FOXM1 co-regulated a specific set of target genes in various metabolic processes and oncogenic signaling pathways. Among them, POLD1, which encodes the catalytic subunit of DNA polymerase δ, was ranked as the top downstream target co-regulated by CENPF and FOXM1. POLD1 expression was positively correlated with that of FOXM1 and CENPF in HCCs. In addition, POLD1 expression was significantly upregulated in HCC tumors. Functionally, *in vivo* orthotopic injection model showed that stable knockdown of POLD1 in HCC cells suppressed tumor incidence and tumorigenicity and had a trend of diminished lung metastasis.

**Conclusion:**

Taken together, our data suggest that CENPF and FOXM1 could synergistically support hepatocarcinogenesis via the regulation of POLD1. CENPF and FOXM1 may represent new vulnerabilities to novel drug-based therapy in HCC.

## Introduction

Hepatocellular carcinoma (HCC) is one of the leading causes of cancer death worldwide (1, 2) and the second and third commonest cancer, respectively, in China and Hong Kong. Indeed, 55% of all new liver cancers worldwide each year occur in China including Hong Kong, due to a high prevalence of hepatitis B viral (HBV) infection (3, 4). It has a poor prognosis and only few effective treatment options are available. Despite years of efforts in studying the molecular mechanism of HCC carcinogenesis, current understanding on this lethal disease is still limited. In a recent study of our group (5), we have utilized whole-transcriptome sequencing technology to perform a differential gene expression (DGE) analysis using the dataset of 50 pairs (tumor and the corresponding non-tumorous liver tissue) of HCC cases from The Cancer Genome Atlas (TCGA). By comparing the gene expression in HCC tumors and their corresponding non-tumorous liver tissues, differentially expressed genes in HCC were identified. Among the 734 differentially expressed genes, CENPF and FOXM1 were listed as the first and third most upregulated genes respectively in HCC. Both FOXM1 and CENPF are crucial for cell-cycle progression, especially in the G2/M phase.

In a study on mitosis regulation and aging, CENPF was demonstrated to be a direct target of the cell cycle master regulator FOXM1 (6). Hence, it is traditionally believed that CENPF is a downstream target of FOXM1 and under the regulation by FOXM1. Interestingly, CENPF and FOXM1 were predicted to be master regulators of prostate cancer malignancy in a cross-species computational analysis by comparing interactomes of human and mice (7), and experimental validation demonstrated that they function synergistically to promote tumor growth by regulating prostate cancer-associated target gene expression profiles. Knockdown of CENPF and FOXM1 synergistically reduced the proliferation of cancer cells and tumor growth in cell-line-derived xenografts. It was further demonstrated that knockdown of CENPF expression reduced the binding of FOXM1 to its targets, suggesting CENPF is required for appropriate genomic binding by FOXM1. Additional data showed that they were co-localized in nucleus and their subcellular co-localization was mutually dependent.

Taken together, multiple lines of evidence imply CENPF and FOXM1 may have novel cooperative roles in regulating the expression of shared target genes. Hence, we postulate that they may have functional synergism in driving hepatocarcinogenesis.

## Materials and Methods

### Quantitative Real-Time Polymerase Chain Reaction

RNA was extracted by Trizol (Thermo Fisher Scientific, Waltham, MA, United States) and cDNA was synthesized by a reverse transcription kit (Thermo Fisher Scientific, Waltham, MA, United States). Quantitative real-time polymerase chain reaction (qRT-PCR) was performed with target-specific TaqMan probes (Thermo Fisher Scientific, Waltham, MA, United States) listed in Supplementary Table 1. The mRNA expression was normalized by the expression of the housekeeping gene *HPRT*.

### Clinical Specimens and Clinicopathological Correlation Analysis

The primary HCC specimens and their corresponding non-tumorous liver tissues of randomly selected 118 human HCC cases were surgically resected from HCC patients in Queen Mary Hospital of Hong Kong between year 1991 and 2017. None of the patients had received therapies before hepatic tumor resection. Total RNA of these 118 pairs clinical specimens were isolated for subsequent qRT-PCR and clinicopathological correlation analysis using SPSS24.0 software, as previously described (8). We did not differentiate into micro- or macrovascular invasion. However, although the venous invasion included both microvascular and macrovascular invasion, mostly it was microvascular invasion. The tumor microsatellites were defined as microscopic or small tumor nodules less than 1 cm in diameter and in close proximity to the main tumor. Direct liver invasion was defined as invasion of the tumor cells into the non-tumorous liver parenchyma without separation by tumor capsule or fibrous layer (9). The use of clinical specimens was approved by the Institutional Review Board of the University of Hong Kong and the Hospital Authority.

### Cell Lines and Culture Conditions

Hepatocellular carcinoma cell line Hep3B (HB-8064) was obtained from the American Type Culture Collection (ATCC) and HCC cell line Huh7 (JCRB0403) was obtained from JCRB Cell Bank. MHCC97L was a gift from Liver Cancer Institute, Fudan University. Hep3B cells were cultured in Minimum Essential Medium (MEM) supplemented with 1mM sodium pyruvate (NaPy). MHCC97L cells were cultured in Dulbecco’s modified Eagle minimal high glucose essential medium (DMEM-HG) supplemented with 1mM NaPy. Huh7 cells were cultured in DMEM-HG media. All cell culture media mentioned above were further supplemented with 10% fetal bovine serum (FBS), 1% penicillin, and 1% streptomycin unless otherwise specified. Cell line cultures were maintained in 37°C and 5% CO_2_ incubator.

Authentication of HCC cell lines used in this study was performed by short tandem repeat (STR) DNA Profiling in March 2018 and no cellular cross-contamination was detected. STR result for MHCC97L is provided in Supplementary Figure 1. Cell cultures were tested negative for Mycoplasma contamination. “Xenome,” utilizing RNA-seq data, estimated a negligible 0.04–0.42% (*n* = 3) for MHCC97L, while 0.15–0.40% for clinical human NTL and HCC samples (*n* = 6) with mouse contamination, thus indicating our MHCC97L cells do not contain cells of murine origin (10). Furthermore, MHCC97L used in this study contains HBV integration in the TERT locus of the genome (8).

### Stable Lentiviral-Based Short-Hairpin RNA Knockdown Cell Models

FOXM1, CENPF, and POLD1 were stably knocked down in MHCC97L HCC cells by the lentiviral-based short-hairpin RNA (shRNA) approach as previously described (11, 12). The oligonucleotide sequences encoding non-targeted control (shNTCC) shRNA and shRNAs that specifically target FOXM1 (shFOXM1), CENPF (shCENPF), and POLD1 (shPOLD1) (Integrated DNA Technologies, Coralville, IA, United States) were summarized in Supplementary Table 2. The forward and reverse oligonucleotides were reannealed to generate shRNA cassettes and each of them was individually cloned into pLKO.1-Puro plasmid with puromycin resistance gene as selection marker. For lentiviral packaging, individual shRNA plasmid was co-transfected with the packaging mix into 293FT cells by lipofectamine 2000 transfection reagent (Invitrogen) at a plasmid to lipofectamine ratio (μg:μL) of 1:2. The viral supernatants were harvested 48 h post-transfection and used to transduce MHCC97L cells. About 1 μg/mL puromycin was applied to the viral-transduced cells for at least 4 days to select for stable knockdown clones. The knockdown efficiencies of FOXM1 and CENPF were examined by RT-qPCR and western blot.

### Western Blot Analysis

Cells were lysed by 6X protein sample buffer (0.35 M Tris-HCl, pH 6.8, 30% glycerol, 20% SDS, 9.3% DTT and 0.05% bromophenol blue) and resolved by SDS-polyacrylamide gel electrophoresis. Immunoblots were incubated with primary antibody rabbit anti-human FOXM1 (D12D5) XP (Cell Signaling Technology, Danvers, MA, United States), rabbit anti-human CENPF (D6X4L) (Cell Signaling Technology, Danvers, MA, United States) and mouse anti-human β-actin (Sigma Aldrich, St. Louis, MO, United States) at 4 C overnight, followed by horseradish peroxidase-labeled anti-rabbit or anti-mouse secondary antibodies (Sigma Aldrich, St. Louis, MO, United States) at room temperature for 2 h. The chemiluminescence signal was detected with the ECL detection system (GE Healthcare, Lafayette, CO, United States).

### Cell Proliferation Assay by Direct Cell Counting

About 2 × 10^4^ cells of each cell line were seeded into each well of multiple 24-well culture plates in triplicates and incubated in a 37°C humidified incubator with 5% CO_2_. Cell growth was assessed by determining the number of cells in each well every 24 h for 4 consecutive days. Direct cell counting was performed using COULTER COUNTER Z1 Cell and Particle Counter (Beckman Coulter).

### Cell Growth Inhibition Assay by XTT Assay

Cell Proliferation II Kit (XTT) (Roche, Basel, Switzerland) was used to determine the percentage of cell growth inhibition according to the manufacturer’s instructions.

### Animal Studies

All animal studies were approved by the Animals (Control of Experiments) Ordinance of Hong Kong and the Committee on the Use of Live Animals in Teaching and Research (CULATR) of the University of Hong Kong (CULATR number: 3848-15), and strictly following the institutional regulations and guidelines. All animal studies were performed on 4 to 6 weeks old BALB/c-nu/nu (nude) athymic male mice, which were provided by the Centre for Comparative Medicine Research of the University of Hong Kong.

### *In vivo* Orthotopic Liver Injection Model

*POLD1* was knocked down in luciferase-labeled MHCC97L (MHCC97L-Luc) HCC cells by shRNA approach. The orthotopic liver injection was performed on 4 to 6 weeks old BALB/c-nu/nu (nude) athymic male mice to assess the metastatic potential of the injected HCC cells as previously described (13). About 1 × 10^6^ of MHCC97L-luc stable cells were resuspended in 15 μL of Matrigel Basement Membrane Matrix (Corning) diluted with serum-free cell culture medium in a 1:1 ratio and then injected into the left lobe of the livers of mice by a 29-gauge needle (Hamilton, Reno, NV, United States). The abdominal wound was sutured after the injection. At 6-week post-injection, bioluminescence imaging of the xenografts was performed. Mice were anesthetized with Pentobarbital at 80 mg/kg, followed by injection of D-luciferin at 100 mg/kg (Perkin-Elmer, Waltham, MA, United States) into the tumor-bearing mice intraperitoneally. Bioluminescence images were acquired by IVIS Spectrum *in vivo* imaging system (Perkin-Elmer, Waltham, MA, United States) to measure the total flux of the bioluminescent signals emitted from the dissected liver tumor xenografts and the distant lung metastases.

### Identification of FOXM1 and CENPF Co-regulated Genes by RNA-Sequencing

RNA-sequencing (RNA-seq) was performed to identify downstream target genes that were co-regulated by FOXM1 and CENPF. FOXM1 and CENPF were either individually silenced or co-silenced in Huh7, Hep3B and MHCC97L using small-interfering RNA (siRNA) approach and subjected to RNA-seq subsequently. Before transfection, 1.5 × 10^5^ per well of cells were seeded onto a 6-well plate (for RNA extraction) and 3.5 × 10^5^ per well of cells were seeded onto a 60-mm plate (for protein extraction) and were incubated in a 37°C humidified incubator with 5% CO_2_ overnight. The siRNAs of the non-target control (NTC), FOXM1 and/or CENPF (Dharmacon, GE Healthcare, Lafayette, CO, United States) listed in Supplementary Table 3 were transfected into the HCC cells with DharmaFECT 1 transfection reagent (Dharmacon, GE Healthcare, Lafayette, CO, United States). The cells were incubated for further 72 h and then subjected to RNA and protein extraction. Real-time PCR and western blot analysis were performed to confirm the knockdown efficiencies of FOXM1 and CENPF. Successful individual and co-knockdown cells were subjected to bioanalyzer analysis (Agilent) for RNA sample quality control followed by RNA-seq analysis (polyA and mRNA) at the Center for PanorOmic Sciences of the University of Hong Kong. The RNA-seq data has been deposited to NCBI SRA (PRJNA800214).

The DGE profiles of FOXM1 and/or CENPF knockdown HCC cell lines were analyzed by edgeR. Furthermore, additional filters were applied to confine the candidate gene list co-regulated by FOXM1 and CENPF based on four criteria: (i) they were differentially expressed with a *p*-value less than 0.05; (ii) with log CPM (counts per million) greater than 1; (iii) with an absolute log fold change greater than 1; and (iv) they were commonly differentially expressed in all 3 HCC cell lines with co-knockdown of FOXM1 and CENPF. Criteria (i)–(iii) applied to DGE analysis using both TCGA and our in-house whole-transcriptome sequencing of HCC clinical cases. To validate with DGE analysis results from the knockdown cell lines, correlation analysis between the candidate genes and FOXM1 and/or CENPF was performed using the TCGA and our in-house whole-transcriptome sequencing cohort of HCC clinical cases (14). The activated and repressed candidate genes that were potentially co-regulated by FOXM1 and CENPF were also subjected to gene set enrichment analysis (15).

## Results

### Expression and Clinical Relevance of FOXM1 and CENPF in Human HCCs

**FIGURE 1:**
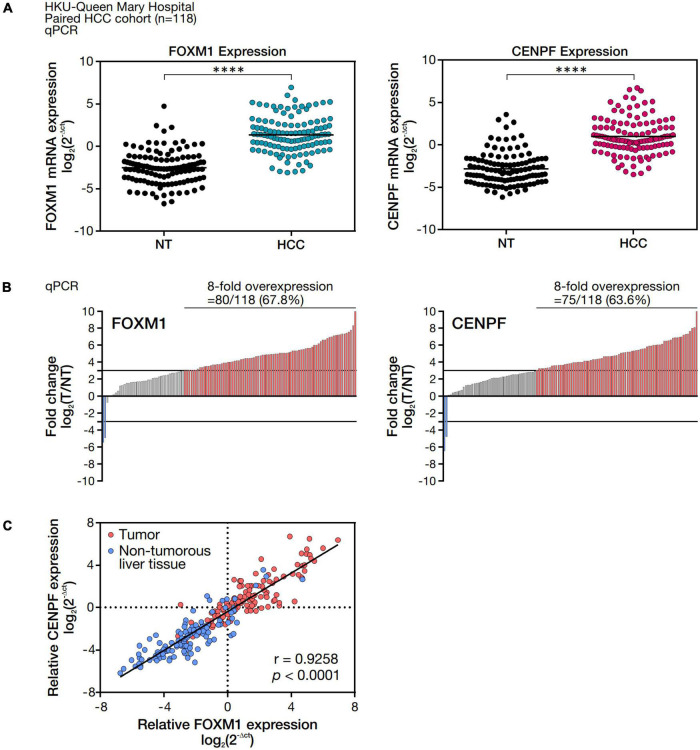
FOXM1 and CENPF mRNA expression level and correlation analysis in HCC tumor and the corresponding non-tumorous liver tissues. **(A)** mRNA expression levels of FOXM1 and CENPF in 118 pairs of HCC and NT samples. mRNA expressions were normalized by housekeeping gene, *HPRT*. **(B)** Waterfall plot showing overall distribution of the fold change of FOXM1 and CENPF mRNA expression in 118 HCC clinical paired samples. **(C)** Pearson correlation analysis between FOXM1 and CENPF relative mRNA expression. Statistical significance was calculated by one-sample *t*-test (^*⁣*⁣**^p < 0.001).

To validate FOXM1 and CENPF expression in our cohort of HCC patients, we examined the abundance of mRNA expression of FOXM1 and CENPF in 118 randomly selected pairs of HCC clinical samples (tumor, HCC, and corresponding non-tumorous liver tissues, NT) by real-time RT-PCR analysis. By comparing the mRNA expression levels between the HCC and NT samples, both FOXM1 and CENPF were shown to be significantly upregulated (^****^p < 0.0001), respectively (Figure 1A). The overall distribution of FOXM1 and CENPF expression in our cohort was displayed in the waterfall plot (Figure 1B). Using a cut-off at 8-fold difference between HCC and NT, 80 of the 118 HCC cases (67.8%) showed an overexpression of FOXM1, and 75 of the 118 HCC cases (63.6%) showed an overexpression of CENPF, whereas only 2 HCC cases (1.69%) had under-expression of either FOXM1 or CENPF. Interestingly, when we compared the relative expression of FOXM1 and CENPF in HCC and NT, respectively, we observed that they were positively correlated with each other (*r* = 0.9258, *p* < 0.0001), and substantially higher expressions in the tumor samples (Figure 1C).

To examine the clinical relevance of FOXM1 and CENPF, either in terms of their expression alone or co-expression, clinicopathological correlation analysis was performed. Using a threshold of 8-fold difference between T and NT, we showed that the overexpression of FOXM1 was positively correlated with the presence of tumor microsatellite formation (*p* = 0.045), whereas the overexpression of CENPF was positively correlated with the presence of venous invasion (*p* = 0.011) and the presence of tumor microsatellite (*p* = 0.010). Remarkably, co-overexpression of FOXM1 and CENPF were positively correlated with the presence of venous invasion (*p* = 0.022) and the presence of tumor microsatellite formation (*p* = 0.009), while the absence of tumor encapsulation (*p* = 0.059) was also suggested but did not reach statistical significance (Table 1).

**TABLE 1:** Clinicopathological correlation analysis of FOXM1 and CENPF.

Clinical parameters		FOXM1 expression (T/NT)			CENPF expression (T/NT)			FOXM1 and CENPF expression (T/NT)		
		≥8-Fold	<8-Fold	*p*-Value	≥8-Fold	<8-Fold	*p*-Value	≥8-Fold	<8-Fold	*p*-Value
Gender	Male	63	28	0.348	60	31	0.223	54	37	0.446
	Female	17	10		15	12		15	12	
Venous invasion	Yes	48	19	0.204	49	18	0.011*	45	22	0.022*
	No	32	19		26	25		24	27	
Tumor encapsulation	Yes	23	15	0.146	20	18	0.057	18	20	0.059
	No	57	22		55	24		51	28	
Tumor microsatellite formation	Yes	48	16	0.045*	47	17	0.010*	44	20	0.009*
	No	31	22		27	26		24	29	
Direct liver invasion	Yes	32	10	0.123	29	13	0.254	27	15	0.225
	No	44	25		42	27		38	31	
Tumor size	>5 cm	53	22	0.221	50	25	0.204	46	29	0.228
	≤5 cm	26	16		24	18		22	20	
Cirrhosis	Yes	35	23	0.066	36	22	0.445	31	27	0.183
	No	45	15		39	21		38	22	
Chronic liver disease	Yes	73	37	0.205	68	42	0.140	62	48	0.084
	No	7	1		7	1		7	1	
Cellular differentiation (Edmonson’s grading)	I–II	34	18	0.403	33	19	0.560	31	21	0.459
	III–IV	45	20		41	24		37	28	
Tumor stage	I/II	25	16	0.195	22	19	0.093	21	20	0.195
	III/IV	53	22		51	24		46	29	

Clinicopathological correlation analysis between the indicated clinical parameters and the individual and co-expression of FOXM1 and CENPF in human HCC (n = 118) at an 8-fold difference cut-off. Statistical analyses were done by SPSS software 24.0. Asterisks (*) indicate statistically significant (p < 0.05). T; HCC tumorous tissue; NT; corresponding non-tumorous liver tissue. P values are shown in bold to emphasize they are < 0.05.

### FOXM1 and CENPF Were Critical for Cell Proliferation

**FIGURE 2:**
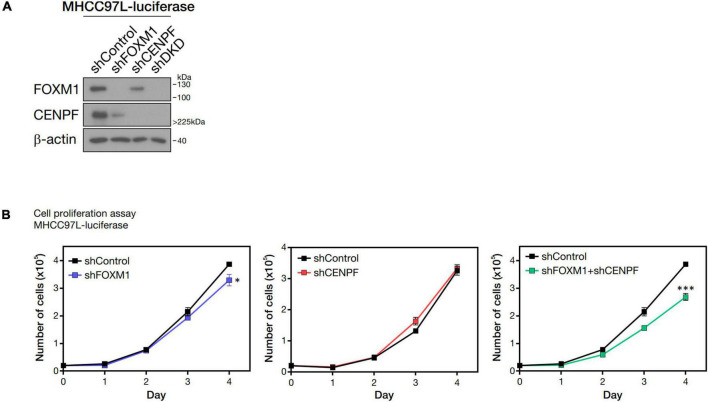
Pro-proliferative role of FOXM1 and CENPF. **(A)** Western blot analysis showing single and double knockdown efficiencies of FOXM1 and CENPF in MHCC97L-Luc cells. **(B)** Representative cell proliferation assay showing the growth curves of HCC cells with single and co-knockdown of FOXM1 and CENPF for four consecutive days. Statistical significance was calculated by one-sample *t*-test (*p < 0.05; ^***^p < 0.001). shDKD, shRNA double knockdown of FOXM1 and CENPF cells.

FOXM1 and CENPF were individually silenced and co-silenced in MHCC97L-Luc HCC cell line by shRNA approach. We confirmed the successful knockdown by Western blot analysis (Figure 2A). To investigate the role of FOXM1 and CENPF in cell proliferation *in vitro*, the established stable knockdown cell lines were analyzed by anchorage-dependent cell proliferation assay. Co-knockdown of FOXM1 and CENPF cells showed the most significantly reduced proliferation rate (*p < 0.05*) (Figure 2B).

### Identification of Commonly Regulated Genes by FOXM1 and CENPF Using RNA-Seq

To investigate the potential synergistic underlying mechanism of FOXM1 and CENPF in HCC, we have established single and co-knockdown FOXM1 and CENPF cell lines using small-interfering RNA (siRNA) transient knockdown approach for subsequent RNA-seq. siRNA knockdown cell lines were established in Huh7, Hep3B and MHCC97L HCC cells. The knockdown efficiencies were confirmed by real-time RT-PCR and western blot analysis (Supplementary Figure 2). Among all, siFOXM1-6, siCENPF-10 and siFOXM1-6/siCENPF-10 exhibited strongest knockdown efficiencies consistently in all 3 HCC cell lines. Thus, they were used for RNA-seq, together with the siRNA control cells.

**FIGURE 3:**
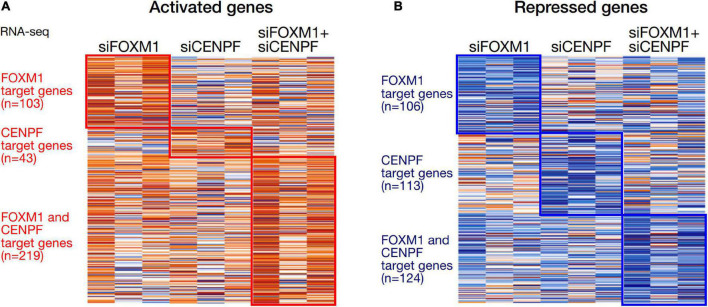
Heatmaps of the differentially expressed genes in HCC cells with knockdown of FOXM1 and CENPF. **(A)** Orange color indicates genes that were activated upon silencing of FOXM1 and/or CENPF. 103 genes were upregulated upon FOXM1 knockdown. About 43 genes were upregulated upon CENPF knockdown. 219 genes were upregulated upon FOXM1 and CENPF co-knockdown. **(B)** Blue color indicates genes that were repressed upon silencing of FOXM1 and/or CENPF. About 106 genes were downregulated upon FOXM1 knockdown. 113 genes were downregulated upon CENPF knockdown. About 124 genes were downregulated upon FOXM1 and CENPF co-knockdown.

We analyzed the expression profiles from the HCC cell lines in which they were individually silenced or co-silenced by DGE analysis. From the DGE analysis across all 3 sets of knockdown cell lines, we have identified 209 genes that were regulated by FOXM1, with 103 being activated and 106 being repressed upon FOXM1 knockdown; 156 genes that were regulated by CENPF, with 43 being activated and 113 being repressed upon CENPF knockdown; and 343 genes that were co-regulated by FOXM1 and CENPF, with 219 being activated and 124 being repressed upon co-knockdown of FOXM1 and CENPF (Figure 3). Among 343 candidate genes that were potentially being co-regulated by FOXM1 and CENPF, we further filtered out 29 genes that were activated and 4 genes that were repressed upon co-knockdown of FOXM1 and CENPF in HCC cells (Supplementary Figure 3).

To elucidate the molecular pathways underlying the synergistic interaction of FOXM1 and CENPF, we subjected the list of activated and repressed candidate genes for gene set enrichment analysis using two databases, including Gene Ontology (biological process) and Reactome Pathway. We found that the differentially expressed genes were mainly enriched in biological pathways associated with various metabolic processes and biological signaling pathways. Notably, these genes were also enriched in several signaling pathways that are associated with tumorigenesis, including insulin growth factor (IGF) and platelet-derived growth factor (PDGF) signaling pathways (Supplementary Figure 4).

**FIGURE 4:**
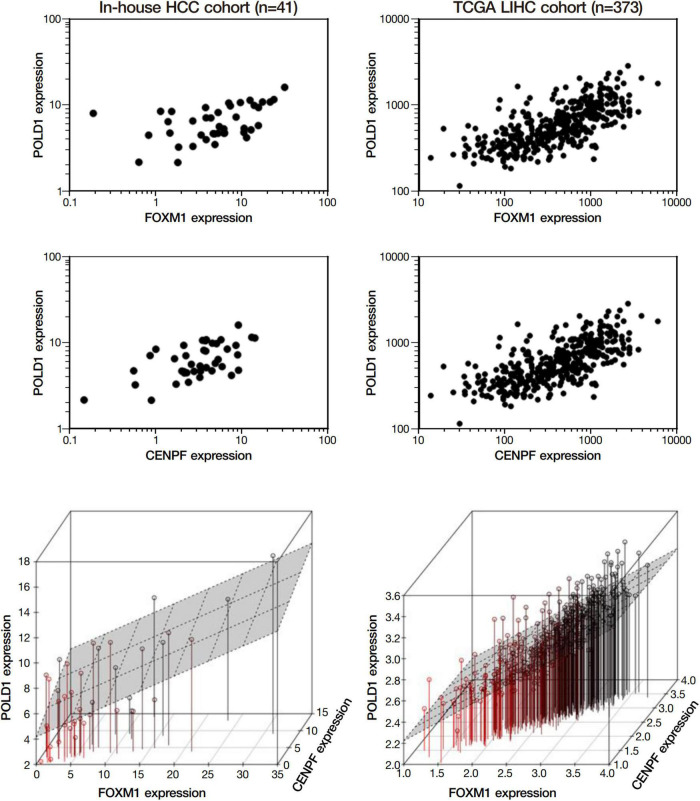
POLD1 was positively correlated with FOXM1 and CENPF. Correlation analysis among the list of repressed candidate genes using our in-house RNA-seq dataset and the TCGA RNA-seq dataset. Both analyses indicated POLD1 was significantly correlated to both FOXM1 and CENPF (*p* < 0.05). Three-dimensional plot showed the correlation between the expressions of FOXM1, CENPF and POLD1 in both datasets.

**FIGURE 5:**
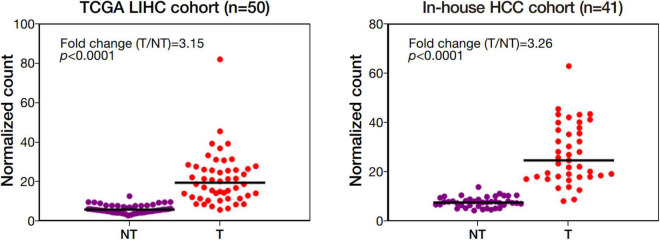
POLD1 was significantly upregulated in human HCC. Differential gene expression analysis of POLD1 in the Cancer Genome Atlas (TCGA) RNA-seq dataset of 50 pairs of human HCC and our in-house RNA-seq dataset of 41 matched pairs of human HCC.

**FIGURE 6:**
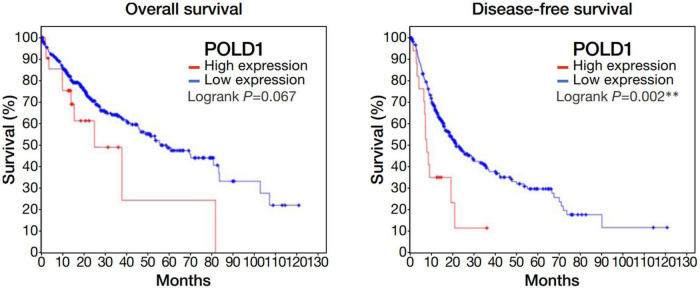
Upregulation of POLD1 in HCC tumors was associated with poorer prognosis in TCGA dataset. Kaplan-Meier curves displayed the overall survival and disease free survival rates of HCC patients with higher POLD1 expression relative to those with lower expression. Higher POLD1 expression was based on a threshold of 2 for the mRNA expression z-scores relative to diploid samples.

Among the 4 repressed gene candidates (Supplementary Figure 3), correlation analysis was performed using our in-house RNA-seq and the TCGA dataset. We found that *POLD1* (DNA Polymerase Delta 1) is positively correlated with both FOXM1 and CENPF, respectively and concurrently, in both RNA-seq datasets (Figure 4). In addition, *POLD1* expression was significantly upregulated in HCC tumor when compared with the corresponding liver tissue in TCGA RNA-seq dataset (fold change = 3.15, *p* < 0.0001) and in our in-house RNA-seq dataset (fold change = 3.26, *p* < 0.0001) (Figure 5). Furthermore, we found that HCC patients with higher expression of *POLD1* had shorter overall survival and disease-free survival rates from the TCGA data analysis (Figure 6).

### Effect of Knockdown of POLD1 in Tumorigenicity and Lung Metastasis in HCC

**FIGURE 7:**
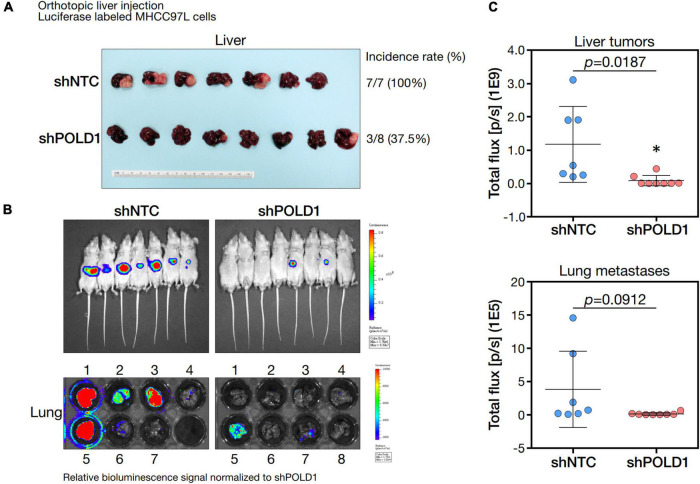
Knockdown of POLD1 in MHCC97L-luc cells reduced tumor incidence, tumor sizes and lung metastases in orthotopic liver injection model. The *in vivo* functional role of POLD1 knockdown was evaluated as compared to the non-target control (NTC). Comparison was based on **(A)** tumorigenicity and **(B,C)** tumor size and lung metastasis.

We knockdown *POLD1* (shPOLD1) in MHCC97L HCC cells by shRNA approach and performed *in vivo* liver orthotopic injection assay to assess the functional role of *POLD1* in liver tumorigenicity and lung metastasis. We showed that shPOLD1 strongly reduced tumor incidence. The tumor sizes (measured by total flux signal) were significantly reduced in mice injected with shPOLD1 HCC cells. We also observed a trend of diminished lung metastasis upon knockdown of POLD1, but the difference could not reach statistical significance (Figure 7).

## Discussion

Development of HCC is a multistep process results from accumulation of mutational events in cancer driver genes. The alterations in driver genes promote oncogenic functions, such as proliferation, survival, cell motility and immune evasion. Moreover, accumulation of genetic and epigenetic alterations could contribute to tumor initiation, progression, metastasis and resistance to therapy (16). The malignant phenotype of cancer entails the dysregulation of cell cycle machinery, which serves as a convergence point for cellular transformation (17).

In this study, we demonstrated the significant roles of FOXM1 and CENPF in hepatocarcinogenesis. First, we found the co-overexpression of FOXM1 and CENPF in HCC was associated with aggressive tumor behavior, including the presence of venous invasion, tumor microsatellite formation, and the absence of tumor encapsulation by clinicopathological correlation analysis. Second, we demonstrated that FOXM1 and CENPF are important for cell growth. Third, our RNA-seq study further delineated that FOXM1 and CENPF co-regulated a set of genes that play essential roles in various metabolic processes and oncogenic signaling pathways. Among all differentially expressed genes between the wild-type HCC cells and FOXM1 and/or CENPF knockdown cell lines, *POLD1*, which encodes for the catalytic subunit of DNA polymerase δ, was ranked as the top downstream target co-regulated by FOXM1 and CENPF.

In addition to the delineation of underlying mechanism of FOXM1 and CENPF in HCC, we have also been able to demonstrate their therapeutic implications in HCC using *in vitro* models. Despite the efficacy of existing molecularly targeted drugs, e.g., sorafenib and regorafenib, there is still an unmet medical need for patients with advanced HCC. With the successful advent of immune checkpoint inhibitors (ICIs), the combo of atezolizumab and bevacizumab has become the standard of care. However, although early clinical outcomes are impressive, a significant proportion of patients do not respond to this regimen. Uncontrolled cell growth is one of the characteristics of cancer, which entails aberrant activities of cell cycle proteins. Upregulation of FOXM1 and CENPF were shown to be crucial for the deregulated cell proliferation in HCC in this study. HCC may be uniquely dependent on FOXM1 and CENPF for cell growth; thus, we speculated that targeting these two cell cycle regulators offer considerable potentials in treating HCC. Thiostrepton is a potential inhibitor of FOXM1 (18, 19) while zoledronic acid is a potential inhibitor of CENPF (20–22). Indeed, we observed thiostrepton and zoledronic acid inhibited HCC cells selectively, as compared to the normal liver cells (Supplementary Figure 5). Further studies are awaited to confirm their potential usage in HCC treatment. It would also be interesting to test these inhibitors in the presence of ICI treatment, for instance in immunocompetent mouse models.

Taken together, our study provided new insight into the underlying synergistic mechanism of FOXM1 and CENPF via the regulation of POLD1, which plays a significant role in HCC progression. Moreover, FOXM1 and CENPF also represent new vulnerabilities to novel drug-based therapy in HCC.

## Data Availability Statement

The datasets presented in this study can be found in online repositories. The names of the repository/repositories and accession number(s) can be found in https://www.ncbi.nlm.nih.gov/bioproject/, PRJNA800214.

## Ethics Statement

The studies involving human participants were reviewed and approved by Institutional Review Board of the University of Hong Kong/Hospital Authority Hong Kong West Cluster. The patients/participants provided their written informed consent to participate in this study. The animal study was reviewed and approved by Committee on the Use of Live Animals in Teaching and Research (CULATR) of the University of Hong Kong.

## Author Contributions

DH and IN: study concept and design. DH, W-LL, L-KC, and IN: acquisition of data, analysis and interpretation of data, and drafting of the manuscript. IN: acquisition of clinical samples. All authors reviewed and approved the final draft of the manuscript.

## Conflict of Interest

The authors declare that the research was conducted in the absence of any commercial or financial relationships that could be construed as a potential conflict of interest.

## Publisher’s Note

All claims expressed in this article are solely those of the authors and do not necessarily represent those of their affiliated organizations, or those of the publisher, the editors and the reviewers. Any product that may be evaluated in this article, or claim that may be made by its manufacturer, is not guaranteed or endorsed by the publisher.

## Supplementary Material

Supplementary Figure 1 Short tandem repeat (STR) DNA profiling of MHCC97L.

Supplementary Figure 2 Transient knockdown efficiencies of FOXM1 and CENPF in Huh7, Hep3B and MHCC97L. **(A)** mRNA expression and **(B)** protein expression of FOXM1 and CENPF upon siRNA transfection.

Supplementary Figure 3 Candidate genes that were co-regulated by FOXM1 and CENPF. Additional filters (differential expression with *p* < 0.05; logCPM > 1; and | logFC| > 1) were used to narrow down the list of candidate genes. Among all, there were 29 genes being activated and 4 being repressed upon co-silencing of FOXM1 and CENPF across Huh7, Hep3B and MHCC97L.

Supplementary Figure 4 Gene set enrichment analysis of the candidate genes co-regulated by FOXM1 and CENPF. The analysis was performed using Gene Ontology (GO) biological processes and Reactome Pathway.

Supplementary Figure 5 Half-maximal inhibitory concentration (IC_50_) of thiostrepton and zoledronic acid in HCC cell and non-tumorigenic liver cells. The percent growth inhibition curve showed the response of the Huh7 HCC cell line and two non-tumorigenic liver cell lines, including MIHA and LO2, to thiostrepton or zoledronic acid treatments at varying dosage. Thio, thiostrepton; ZOL, zoledronic acid.

## References

[1] Sung H Ferlay J Siegel RL Laversanne M Soerjomataram I Jemal A Global cancer statistics 2020: globocan estimates of incidence and mortality worldwide for 36 cancers in 185 countries. *CA Cancer J Clin.* (2021) 71:209–49. 10.3322/caac.21660 33538338

[2] Llovet JM Kelley RK Villanueva A Singal AG Pikarsky E Roayaie S Hepatocellular carcinoma. *Nat Rev Dis Primers.* (2021) 7:6. 10.1038/s41572-020-00240-3 33479224 PMC13537433

[3] Ho DW Lo RC Chan LK Ng IO. Molecular pathogenesis of hepatocellular carcinoma. *Liver Cancer.* (2016) 5:290–302. 10.1159/000449340 27781201 PMC5075835

[4] Ho DW Lyu X Ng IO. Viral integration detection strategies and a technical update on virus-clip. *Biocell.* (2021) 45:1495–500. 10.32604/biocell.2021.017227

[5] Ho DW Kai AK Ng IO. TCGA whole-transcriptome sequencing data reveals significantly dysregulated genes and signaling pathways in hepatocellular carcinoma. *Front Med.* (2015) 9:322–30. 10.1007/s11684-015-0408-9 26276037

[6] Laoukili J Kooistra MR Bras A Kauw J Kerkhoven RM Morrison A FoxM1 is required for execution of the mitotic programme and chromosome stability. *Nat Cell Biol.* (2005) 7:126–36. 10.1038/ncb1217 15654331

[7] Aytes A Mitrofanova A Lefebvre C Alvarez MJ Castillo-Martin M Zheng T Cross-species regulatory network analysis identifies a synergistic interaction between FOXM1 and CENPF that drives prostate cancer malignancy. *Cancer Cell.* (2014) 25:638–51. 10.1016/j.ccr.2014.03.017 24823640 PMC4051317

[8] Sze KM Ho DW Chiu YT Tsui YM Chan LK Lee JM Hepatitis B virus-telomerase reverse transcriptase promoter integration harnesses host ELF4, resulting in telomerase reverse transcriptase gene transcription in hepatocellular carcinoma. *Hepatology.* (2021) 73:23–40. 10.1002/hep.31231 32170761 PMC7898544

[9] Ng IO Lai EC Ng MM Fan ST. Tumor encapsulation in hepatocellular carcinoma. A pathologic study of 189 cases. *Cancer.* (1992) 70:45–9. 10.1002/1097-0142(19920701)70:1<45::aid-cncr2820700108>3.0.co;2-7 1318778

[10] Conway T Wazny J Bromage A Tymms M Sooraj D Williams ED Xenome–a tool for classifying reads from xenograft samples. *Bioinformatics.* (2012) 28:i172–8. 10.1093/bioinformatics/bts236 22689758 PMC3371868

[11] Ho DWH Chan LK Chiu YT Xu IMJ Poon RTP Cheung TT TSC1/2 mutations define a molecular subset of HCC with aggressive behaviour and treatment implication. *Gut.* (2017) 66:1496–506. 10.1136/gutjnl-2016-312734 27974549 PMC5530480

[12] Ho DW Tsui YM Sze KM Chan LK Cheung TT Lee E Single-cell transcriptomics reveals the landscape of intra-tumoral heterogeneity and stemness-related subpopulations in liver cancer. *Cancer Lett.* (2019) 459:176–85. 10.1016/j.canlet.2019.06.002 31195060

[13] Ma W Ho DW Sze KM Tsui YM Chan LK Lee JM APOBEC3B promotes hepatocarcinogenesis and metastasis through novel deaminase-independent activity. *Mol Carcinog.* (2019) 58:643–53. 10.1002/mc.22956 30575099

[14] Ho DW Tsui YM Chan LK Sze KM Zhang X Cheu JW Single-cell RNA sequencing shows the immunosuppressive landscape and tumor heterogeneity of HBV-associated hepatocellular carcinoma. *Nat Commun.* (2021) 12:3684. 10.1038/s41467-021-24010-1 34140495 PMC8211687

[15] Ho DW Ng IO. uGPA: unified gene pathway analyzer package for high-throughput genome-wide screening data provides mechanistic overview on human diseases. *Clin Chim Acta.* (2015) 441:105–8. 25549900 10.1016/j.cca.2014.12.028

[16] Llovet JM Zucman-Rossi J Pikarsky E Sangro B Schwartz M Sherman M Hepatocellular carcinoma. *Nat Rev Dis Primers.* (2016) 2:16018. 10.1038/nrdp.2016.18 27158749

[17] Williams GH Stoeber K. The cell cycle and cancer. *J Pathol.* (2012) 226:352–64. 21990031 10.1002/path.3022

[18] Hegde NS Sanders DA Rodriguez R Balasubramanian S. The transcription factor FOXM1 is a cellular target of the natural product thiostrepton. *Nat Chem.* (2011) 3:725–31. 10.1038/nchem.1114 21860463

[19] Kwok JM Myatt SS Marson CM Coombes RC Constantinidou D Lam EW. Thiostrepton selectively targets breast cancer cells through inhibition of forkhead box M1 expression. *Mol Cancer Ther.* (2008) 7:2022–32. 10.1158/1535-7163.MCT-08-0188 18645012

[20] Brown HK Ottewell PD Coleman RE Holen I. The kinetochore protein Cenp-F is a potential novel target for zoledronic acid in breast cancer cells. *J Cell Mol Med.* (2011) 15:501–13. 10.1111/j.1582-4934.2009.00995.x 20015195 PMC3922372

[21] Mi YJ Gao J Xie JD Cao JY Cui SX Gao HJ Prognostic relevance and therapeutic implications of centromere protein F expression in patients with esophageal squamous cell carcinoma. *Dis Esophagus.* (2013) 26:636–43. 10.1111/dote.12002 23163484

[22] Cao JY Liu L Chen SP Zhang X Mi YJ Liu ZG Prognostic significance and therapeutic implications of centromere protein F expression in human nasopharyngeal carcinoma. *Mol Cancer.* (2010) 9:237. 10.1186/1476-4598-9-237 20828406 PMC2944187

