# Supplementary material for: Investigation of Functional Synergism of CENPF and FOXM1 Identifies POLD1 as Downstream Target in Hepatocellular Carcinoma
Source: Front Med (Lausanne). 2022 Jul 5;9:860395. doi: 10.3389/fmed.2022.860395 (PMC9295863; doi:10.3389/fmed.2022.860395)

Supplementary Figure 1

Short tandem repeat (STR) DNA profiling of MHCC97L

DNA Marker	MHCC97L[4]	MHCC97L (L-171218744P)
AMEL	X, Y	X, Y
CSF1PO	11, 13	11, 13
D13S317	8	8
D16S539	12	12
D5S818	12,13	12,13
D7S820	10	10
TH01	9	9
TPOX	8	8
vWA	14	14
D18S51	--	13, 22
D21S11	--	31.2
D3S1358	--	15, 16
D8S1179	--	12, 13
FGA	--	21, 24
Penta D	--	8, 9
Penta E	--	11, 17
	**Number of shared alleles**	12
	**Total number of alleles in the reference profile**	12
	**Percent match**	100%

Supplementary Figure 2

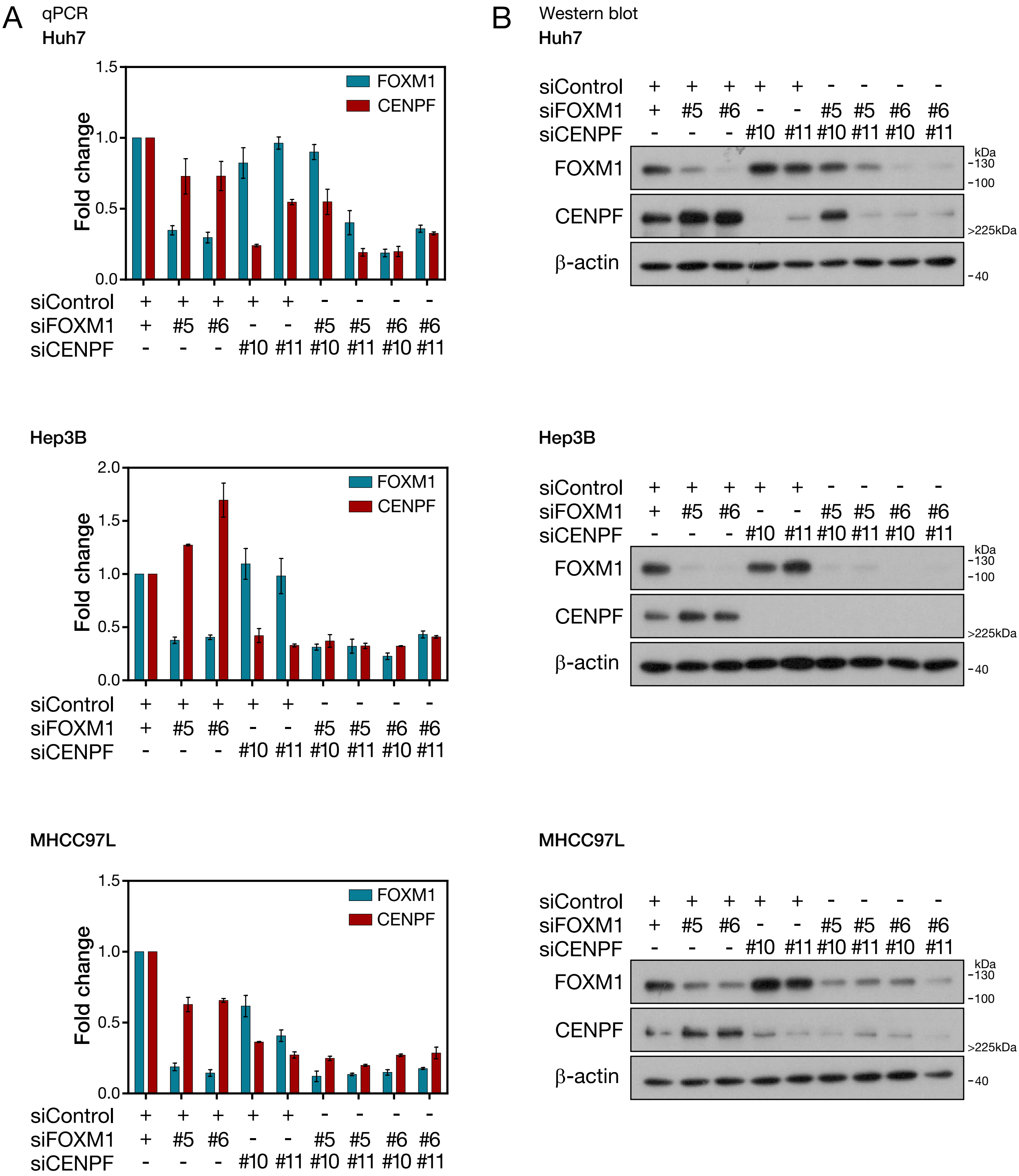

Supplementary Figure 3

RNA-seq

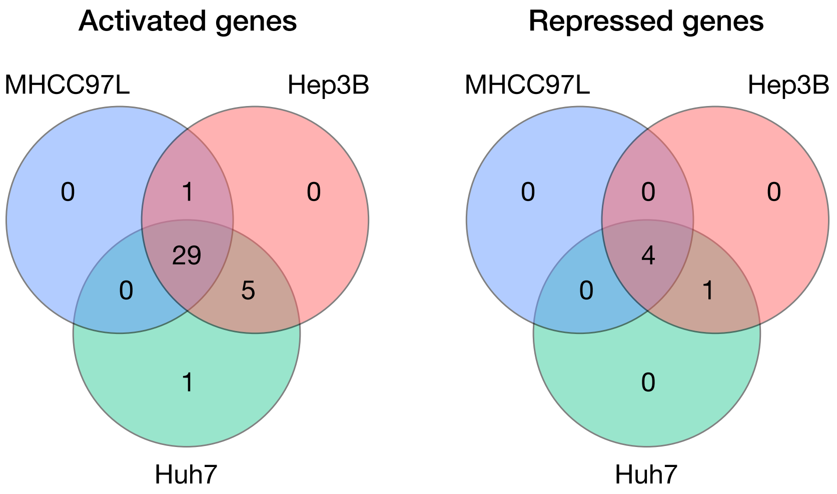

Activated genes (n=29)		
ERLIN1	FLJ23867	NPC1L1
C1RL	LUM	LARP1B
CAT	ACSM2B	FABP1
ACSL5	AIG1	HAAO
LRG1	PBLD	PLG
SULT1E1	AGXT2	MBL2
C1orf168	HMGCL	ANKS4B
SULT1A1	S100A9	ORM2
USH1C	SORL1	APOC3
INHBC	GIPC2	

Repressed genes (n=4)
MDK
POLD1
CBX2
TMEM132A

Supplementary Figure 4

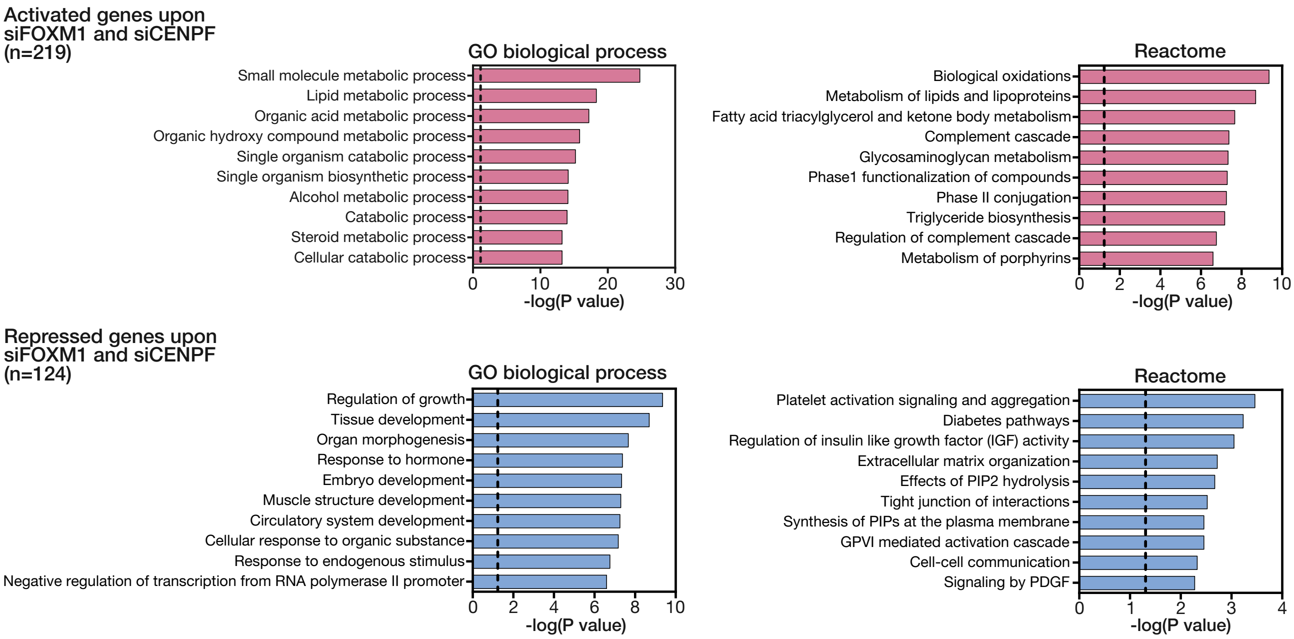

Supplementary Figure 5

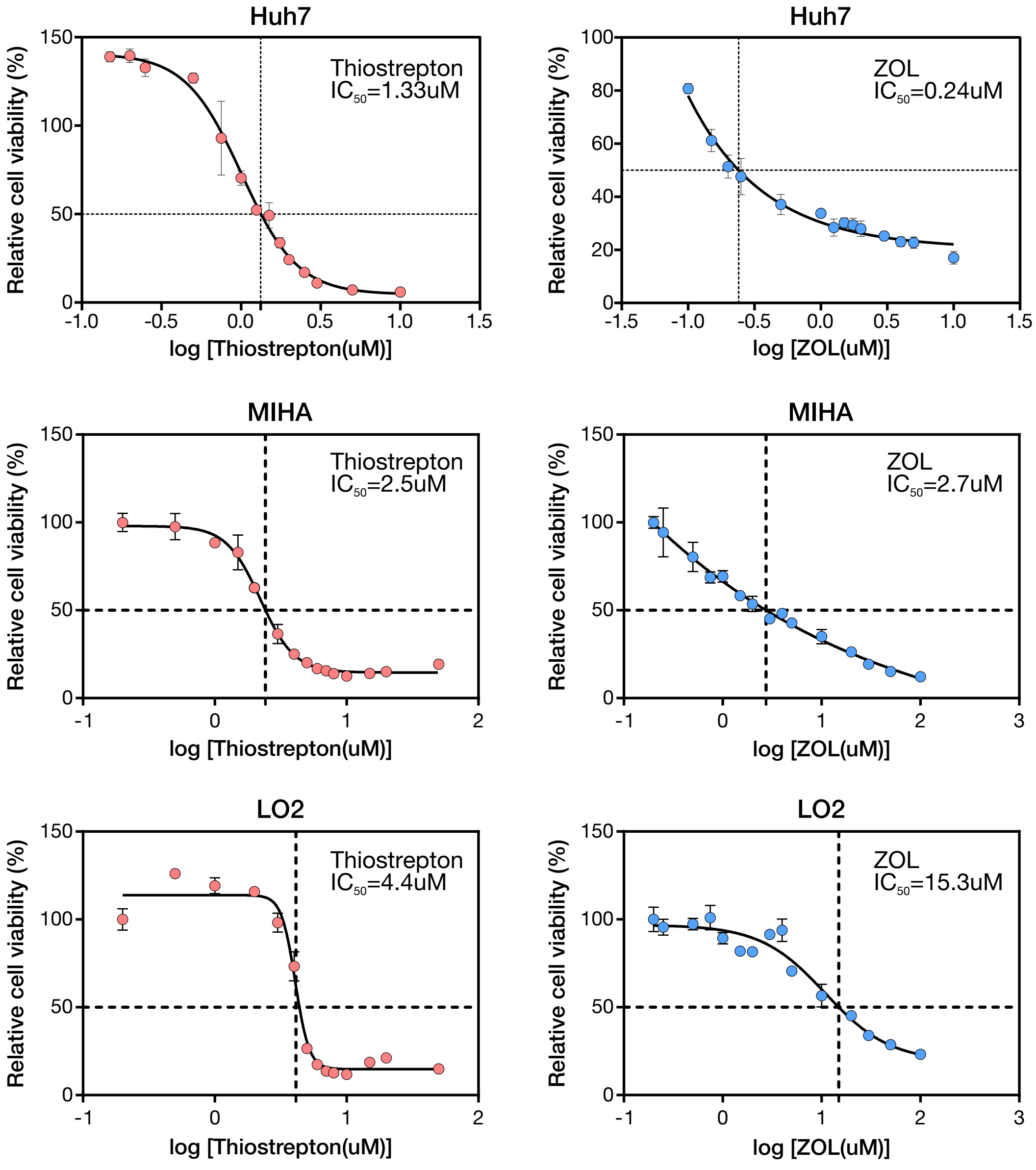

Supplement: Supplementary Figure 1 — Short tandem repeat (STR) DNA profiling of MHCC97L. [file Data_Sheet_2.pdf]
