# Supplementary material for: Investigation of Functional Synergism of CENPF and FOXM1 Identifies POLD1 as Downstream Target in Hepatocellular Carcinoma
Source: Front Med (Lausanne). 2022 Jul 5;9:860395. doi: 10.3389/fmed.2022.860395 (PMC9295863; doi:10.3389/fmed.2022.860395)
Supplement: Supplementary file 2 [file Data_Sheet_1.PDF]

Supplementary Table 1. TaqMan probes used in this study.

Target gene	Assay ID	Reporter dye
FOXM1	Hs01073586_m1	FAM
CENPF	Hs01118845_m1	FAM
HPRT	Hs00177914_m1	VIC

Supplementary Table 2. shRNA targeting sequences used in this study.

shRNA clones	shRNA sequence
shNTC S	CCGGCAACAAGATGAAGAGCACCAACTCGAGTTG GTGCTCTTCATCTTGTTGTTTTTG
shNTC AS	AATTCAAAAACAACAAGATGAAGAGCACCAACTC GAGTTGGTGCTCTTCATCTTGTTG
shFOXM1 S	CCGGGCCCAACAGGAGTCTAATCAACTCGAGTTGA TTAGACTCCTGTTGGGCTTTTTG
shFOXM1 AS	AATTCAAAAAGCCCAACAGGAGTCTAATCAACTCG AGTTGATTAGACTCCTGTTGGGC
shCENPF S	CCGGGCGAGTCAGATCAAGGAGAATCTCGAGATTC TCCTTGATCTGACTCGCTTTTTG
shCENPF AS	AATTCAAAAAGCGAGTCAGATCAAGGAGAATCTC GAGATTCTCCTTGATCTGACTCGC
shPOLD1 S	CCGGCCTGGCACTGATGGAGGAGATCTCGAGATCT CCTCCATCAGTGCCAGGTTTTTG
shPOLD1 AS	AATTCAAAAACCTGGCACTGATGGAGGAGATCTCG AGATCTCCTCCATCAGTGCCAGG

Supplementary Table 3. siRNA reagents for transient knockdown clones.

Gene target	Catalog number
Non-targeting control (NTC)	D-001810-01-05
FOXM1	LQ-009762-00-0002
CENPF	LQ-003253-00-0002
